# Supplementary material for: Empirical methods for controlling false positives and estimating confidence in ChIP-Seq peaks
Source: BMC Bioinformatics. 2008 Dec 5;9:523. doi: 10.1186/1471-2105-9-523 (PMC2628906; doi:10.1186/1471-2105-9-523)
Supplement: Additional File 1 — A variety of html documents from the USeq web site detailing the available applications, their best usage, output file type descriptions, command line menus, etc. [file 1471-2105-9-523-S1.zip › USeqUserGuides/JavaDoc.zip!/JavaDoc/trans/anno/AnnotateRegions.html]

AnnotateRegions


|  |  |  |
| --- | --- | --- |
| **Overview** \| **Package** \| **Class** \| **Use** \| **Tree** \| **Deprecated** \| **Index** \| **Help** | |  |
| PREV CLASS   **NEXT CLASS** | **FRAMES**    **NO FRAMES**     **All Classes** |  |
| SUMMARY: NESTED \| FIELD \| CONSTR \| METHOD | DETAIL: FIELD \| CONSTR \| METHOD |  |


---


## trans.anno Class AnnotateRegions

```
java.lang.Object
  trans.anno.AnnotateRegions
```

---

``` public class AnnotateRegions extends java.lang.Object ```

For annotating Intervals or binding regions with gene information, proximity, neighbors, and bias based on random trials.
Specific for dmel release 4.0 gff3. Each gff3 file needs careful reworking to get it into a form for making gene models.

**See Also:**: `AnnotateRegionsWithGeneList`

---

**Constructor Summary**

`AnnotateRegions(java.lang.String[] args)`


| **Method Summary** | |
| --- | --- |
| `static void` | `compareBindingRegionsVsGeneGrps(GeneGroup[] geneGroups, BindingRegion[] bindingRegions)`             Does a complete scan, could be optimized. |
| `static int[]` | `countGenes(BindingRegion[] br)`             Returns the number of genes where the binding region is on the 5' end and the number of genes where the binding region is on the 3' end of the respective gene, the number of genes that overlap a binding region on their 5' end and 3' end, lastly the number of regions entirely contained by a gene, the number of binding regions with neighbors, the number of regions with no neighbors as defined by the neighborhood, the number of regions in non coding DNA, the number of regions in coding DNA, the number of regions that overlap coding and nonCoding DNA |
| `static int` | `countNumberBindingRegionsWithNeighbors(BindingRegion[] br)` |
| `static int` | `countNumberNeighbors(BindingRegion[] br)` |
| `static java.util.ArrayList` | `extractCGNames(java.util.ArrayList geneGroups)`             Extracts the names of each gene group returning an ArrayList of Strings. |
| `static GeneGroup[]` | `filterGeneGroups(GeneGroup[] genes, java.util.HashSet cgNames)`             Returns an array of GeneGroup whose names were found in the Hash |
| `int` | `findDistanceToATG(BindingRegion br, GeneGroup gp)`             Finds the distance to conservative estimate of an ATG, returns 0 if overlaps. |
| `int` | `findDistToClosestATG(BindingRegion br)`             Finds the distance to the closest ATG translation start site. |
| `int` | `findDistToClosestATG(BindingRegion br, java.util.ArrayList geneGroups)` |
| `int` | `findDistToClosestTranscript(BindingRegion br)`             Finds the distance to the closest ATG translation start site. |
| `int` | `findDistToClosestTranscript(BindingRegion br, java.util.ArrayList geneGroups)` |
| `int` | `findDistToClosestTranscript(BindingRegion br, GeneGroup gp)`             Finds the distance to conservative estimate of start of first exon, returns 0 if overlaps. |
| `static void` | `main(java.lang.String[] args)` |
| `static BindingRegion[]` | `makeRandomBindingRegions(BindingRegion[] br, java.util.HashMap chromLengths, int sizeNeighborhood)`             For each binding region this will make another binding region from the same chromosome with the same length, yet at a random location. |
| `static boolean` | `overlap(java.util.ArrayList ints, int startRegion, int endRegion)`             Tests whether any startEnd int[] in the ArrayList of ints ovelaps a region defined by the startRegion and endRegion. |
| `static BindingRegion[]` | `parseIntervalFile(java.io.File intervalFile, int sizeNeighborhood)`             Attempts to fetch a serialized array of Interval[], then sorts/ ranks the intervals by the median ratio of the sub window. |
| `static BindingRegion[]` | `parsePicksFile(java.io.File picksFile, int sizeNeighborhood)` |
| `void` | `printDistToClosestATGAndTranscript(BindingRegion[] br)`             Prints rank, chrom, start, stop, distance to closest ATG, to closest transcript start. |
| `static void` | `printDocs()` |
| `void` | `processArgs(java.lang.String[] args)`             This method will process each argument and assign new varibles |

**Methods inherited from class java.lang.Object**

`equals, getClass, hashCode, notify, notifyAll, toString, wait, wait, wait`

**Constructor Detail**

### AnnotateRegions

```
public AnnotateRegions(java.lang.String[] args)
```


**Method Detail**

### makeRandomBindingRegions

```
public static BindingRegion[] makeRandomBindingRegions(BindingRegion[] br,
                                                       java.util.HashMap chromLengths,
                                                       int sizeNeighborhood)
```

:   For each binding region this will make another binding region from the same chromosome with the
    same length, yet at a random location.

---


### printDistToClosestATGAndTranscript

```
public void printDistToClosestATGAndTranscript(BindingRegion[] br)
```

:   Prints rank, chrom, start, stop, distance to closest ATG, to closest transcript start.

---


### findDistToClosestATG

```
public int findDistToClosestATG(BindingRegion br)
```

:   Finds the distance to the closest ATG translation start site.

---


### findDistToClosestATG

```
public int findDistToClosestATG(BindingRegion br,
                                java.util.ArrayList geneGroups)
```

---


### findDistToClosestTranscript

```
public int findDistToClosestTranscript(BindingRegion br)
```

:   Finds the distance to the closest ATG translation start site.

---


### findDistToClosestTranscript

```
public int findDistToClosestTranscript(BindingRegion br,
                                       java.util.ArrayList geneGroups)
```

---


### findDistanceToATG

```
public int findDistanceToATG(BindingRegion br,
                             GeneGroup gp)
```

:   Finds the distance to conservative estimate of an ATG, returns 0 if overlaps.

---


### findDistToClosestTranscript

```
public int findDistToClosestTranscript(BindingRegion br,
                                       GeneGroup gp)
```

:   Finds the distance to conservative estimate of start of first exon, returns 0 if overlaps.

---


### countNumberNeighbors

```
public static int countNumberNeighbors(BindingRegion[] br)
```

---


### countNumberBindingRegionsWithNeighbors

```
public static int countNumberBindingRegionsWithNeighbors(BindingRegion[] br)
```

---


### countGenes

```
public static int[] countGenes(BindingRegion[] br)
```

:   Returns the number of genes where the binding region is on the 5' end and
    the number of genes where the binding region is on the 3' end of the respective gene,
    the number of genes that overlap a binding region on their 5' end and 3' end, lastly
    the number of regions entirely contained by a gene,
    the number of binding regions with neighbors,
    the number of regions with no neighbors as defined by the neighborhood,
    the number of regions in non coding DNA,
    the number of regions in coding DNA,
    the number of regions that overlap coding and nonCoding DNA

    :   **Returns:**: int[9] {num 5', num 3', overlap 5', overlap 3', contained, no neighbors, non coding, coding, overlap coding and non coding}

---


### overlap

```
public static boolean overlap(java.util.ArrayList ints,
                              int startRegion,
                              int endRegion)
```

:   Tests whether any startEnd int[] in the ArrayList of ints ovelaps a region defined by the
    startRegion and endRegion. Assumes start is always <= end.

---


### extractCGNames

```
public static java.util.ArrayList extractCGNames(java.util.ArrayList geneGroups)
```

:   Extracts the names of each gene group returning an ArrayList of Strings.

---


### compareBindingRegionsVsGeneGrps

```
public static void compareBindingRegionsVsGeneGrps(GeneGroup[] geneGroups,
                                                   BindingRegion[] bindingRegions)
```

:   Does a complete scan, could be optimized.

---


### parsePicksFile

```
public static BindingRegion[] parsePicksFile(java.io.File picksFile,
                                             int sizeNeighborhood)
```

---


### parseIntervalFile

```
public static BindingRegion[] parseIntervalFile(java.io.File intervalFile,
                                                int sizeNeighborhood)
```

:   Attempts to fetch a serialized array of Interval[], then sorts/ ranks the intervals by the median
    ratio of the sub window. It then uses it to build an array of BindingRegion.
    Will return null if it cannot fetch an Interval[].

---


### printDocs

```
public static void printDocs()
```

---


### processArgs

```
public void processArgs(java.lang.String[] args)
```

:   This method will process each argument and assign new varibles

---


### filterGeneGroups

```
public static GeneGroup[] filterGeneGroups(GeneGroup[] genes,
                                           java.util.HashSet cgNames)
```

:   Returns an array of GeneGroup whose names were found in the Hash

---


### main

```
public static void main(java.lang.String[] args)
```


---


|  |  |  |
| --- | --- | --- |
| **Overview** \| **Package** \| **Class** \| **Use** \| **Tree** \| **Deprecated** \| **Index** \| **Help** | |  |
| PREV CLASS   **NEXT CLASS** | **FRAMES**    **NO FRAMES**     **All Classes** |  |
| SUMMARY: NESTED \| FIELD \| CONSTR \| METHOD | DETAIL: FIELD \| CONSTR \| METHOD |  |


---
